# Optimization of Advanced Live-Cell Imaging through Red/Near-Infrared Dye Labeling and Fluorescence Lifetime-Based Strategies

**DOI:** 10.3390/ijms222011092

**Published:** 2021-10-14

**Authors:** Magalie Bénard, Damien Schapman, Christophe Chamot, Fatéméh Dubois, Guénaëlle Levallet, Hitoshi Komuro, Ludovic Galas

**Affiliations:** 1 Normandie University, UNIROUEN, INSERM, PRIMACEN, 76000 Rouen, France; magalie.benard@univ-rouen.fr (M.B.); damien.schapman@univ-rouen.fr (D.S.); 2 INSERM, 76000 Rouen, France; christophe.chamot@inserm.fr; 3 Normandie University, UNICAEN, CEA, CNRS, ISTCT/CERVOxy Group, GIP CYCERON, 14000 Caen, France; fatemeh.dubois@unicaen.fr (F.D.); guenaelle.levallet@unicaen.fr (G.L.); 4 Department of Pathology, CHU de Caen, 14033 Caen, France; 5 Department of Neurosciences, Lerner Research Institute, Cleveland, OH 44195, USA; KOMURO@ccf.org

**Keywords:** live-cell imaging, sample preservation, fluorescence lifetime, confocal, FLIM, STED, FLIM-STED, τ separation

## Abstract

Fluorescence microscopy is essential for a detailed understanding of cellular processes; however, live-cell preservation during imaging is a matter of debate. In this study, we proposed a guide to optimize advanced light microscopy approaches by reducing light exposure through fluorescence lifetime (τ) exploitation of red/near-infrared dyes. Firstly, we characterized key instrumental elements which revealed that red/near-infrared laser lines with an 86x (Numerical Aperture (NA) = 1.2, water immersion) objective allowed high transmission of fluorescence signals, low irradiance and super-resolution. As a combination of two technologies, i.e., vacuum tubes (e.g., photomultiplier) and semiconductor microelectronics (e.g., avalanche photodiode), type S, X and R of hybrid detectors (HyD-S, HyD-X and HyD-R) were particularly adapted for red/near-infrared photon counting and τ separation. Secondly, we tested and compared lifetime-based imaging including coarse τ separation for confocal microscopy, fitting and phasor plot analysis for fluorescence lifetime microscopy (FLIM), and lifetimes weighting for enhanced stimulated emission depletion (STED) nanoscopy, in light of red/near-infrared multiplexing. Mainly, we showed that the choice of appropriate imaging approach may depend on fluorochrome number, together with their spectral/lifetime characteristics and STED compatibility. Photon-counting mode and sensitivity of HyDs together with phasor plot analysis of fluorescence lifetimes enabled the flexible and fast imaging of multi-labeled living H28 cells. Therefore, a combination of red/near-infrared dyes labeling with lifetime-based strategies offers new perspectives for live-cell imaging by enhancing sample preservation through acquisition time and light exposure reduction.

## 1. Introduction

As previously described [1], successful fluorescence live-cell imaging is linked to the Probe–Sample–Instrument triad, with particular focus on the brightness, photostability and specificity of fluorescent tools as well as on instrumental performance. In addition, multiplexing with fluorescent dyes is a challenge with respect to the possibility of long-term acquisition, spectral overlapping and light exposure leading potentially to phototoxicity and artefacts [2]. Therefore, combining new technological developments with innovative experimental strategies is necessary in order to preserve multi-labeled living cells during high- and super-resolution imaging.

During the past decade, progress in chemistry has led to the development of red/near-infrared organic dyes, including the rhodamines and the Alexa Fluor families [1,3,4,5]. Thanks to low-energy excitation wavelength in the red/near-infrared spectrum, not only is cell preservation is improved but also cell autofluorescence is reduced during time-lapses [1,6]. Among the numerous red/near-infrared dyes some present interesting characteristics for multiplexing or super-resolution imaging, including stimulated emission depletion (STED) nanoscopy [6,7,8]. In particular silicon rhodamine probes (SiR) have been recently proposed for DNA, cytoskeleton proteins, mitochondria cristae and lysosome staining [9,10,11]. In addition, for detection of relative mitochondria membrane potential, red or deep red MitoTrackers are frequently used [12,13], while wheat germ agglutin (WGA) coupled with Alexa Fluor 633/647 or MemBright 5 to 7.5 reveal the plasma membrane [14,15]. Nile Red may also be an interesting marker for lipids, cell membrane and lysosomes [8].

However, the risk of overlapping is important when multiplexing is performed with dyes exhibiting closed spectral properties. In this regard, an alternative to fluorescence spectral separation is fluorescence lifetime (τ) separation. Fluorescence lifetime is an intrinsic property of a fluorophore that does not depend on wavelength excitation or duration of light exposure and is not affected by photobleaching [16,17]. In contrast, viscosity, temperature, and pH may influence the lifetime of fluorescent dyes [16,17]. The fluorescence lifetime of small organic fluorophores varies from 0.1 to 20 ns when measured through Time Correlated Single Photon Counting (TCSPC) [15]. TCSPC packages are now available through user-friendly solutions, leading to democratization of Fluorescence Lifetime Imaging Microscopy (FLIM) [18,19]. FLIM is therefore a potential technique for multiplexing and monitoring the molecular environment of fluorophores [20]. In addition, small organic fluorophores have also been used in FLIM-Förster resonance energy transfer (FRET) experiments, in particular for cell trafficking and endocytosis monitoring [16].

Another challenge in live-cell imaging is super-resolution microscopy with one or several colors. There are many approaches, including stimulated emission depletion (STED), structured illumination microscopy (SIM), photoactivation localization microscopy (PALM), stochastic optical reconstruction microscopy (STORM), and universal point accumulation imaging in the nanoscale topography (uPAINT), with respective limitations in terms of labeling, time and spatial resolutions [7,21]. Here, we will only focus on STED nanoscopy that requires suitable fluorescent dyes such as red/near-infrared Alexa Fluor, Atto and STAR. For live-cell studies with STED nanoscopy, two issues should be considered. Firstly, relatively high laser powers are usually needed; however, reduction can be obtained when a pulsed excitation beam and time-gated detection are used [22,23]. Secondly, photobleaching is a limiting parameter for long time-lapse because fluorescent molecules undergo a large number of exciting/de-exciting cycles [24]. Therefore, depletion with wavelengths in near-infrared (775 nm) or with pulsed infrared lasers is recommended for live-cell imaging [25]. Another possible solution is cell-labeling with exchangeable organic fluorophores such as Nile Red, which guarantees the removal of photobleached fluorophores and their replacement by intact fluorophores circumventing the bleaching limitations of STED nanoscopy [8].

The first aim of this work was to optimize the instrumental configuration for live-cell imaging by characterizing the key elements of our commercial confocal/FLIM/STED system; namely, the power and stability of the light source; the uniformity of the field of illumination, the light transmission, and the irradiance through high-resolution objectives; and the spectral sensitivity and the fluorescence lifetime measurement capacities of last-generation detectors. Then, we compared fluorescence lifetime-based strategies, including coarse and fine τ separation for red/near-infrared dyes multiplexing in living cells. In order to reduce the time of exposure and acquisition, we also developed new protocols for double-labeled live-cell imaging through STED nanoscopy. In particular, we have improved contrast and resolution during live-cell FLIM-STED by using a lifetime weighting process and background noise removal.

## 2. Results

### 2.1. Optimization of Instrumental Configuration for Live-Cell Imaging

For robust time-lapse, power stability during live-cell imaging is necessary to avoid any misinterpretation. The White Light Laser (WLL) source used here for confocal microscopy, FLIM and STED nanoscopy was a pulsed supercontinuum laser that irradiates from 440 to 790 nm. Measurements of laser power have been performed along the WLL spectrum at 448, 488, 543, 594, 638, 685 and 730 nm without any objective prior detection (Figure S1). The choice of laser lines was consistent with the presence of notch filters in the beam path that strongly reduced possible detection of excitation light by detectors. At 100% WLL power and 100% acousto-optic tunable filter (AOTF), maximum laser power (533 ± 56 µW) was observed at 594 nm while weaker one (127 ± 12 µW) was detected at 448 nm (Figure S1A). Laser powers in the red range of the WLL spectrum (638, 685 and 730 nm) were between 225 µW and 375 µW (Figure S1A). Analyses of laser power variations during short (1 s every 10 min) and mid (5 s every 60 min) time recording revealed very good stability of the WLL wavelengths (Figures S1B,C).

The characteristics of microscope objectives are also key parameters for the improvement of live-cell imaging conditions. In this context, transmission values of 86× (water immersion, Numerical Aperture (NA) = 1.2, Working Distance (WD) = 300 µm), 93× (glycerol immersion, NA = 1.3, WD = 300 µm) and 100× (oil immersion, NA = 1.4, WD = 100 µm) objectives have been studied with regard to excitation wavelengths at maximum laser power. Whatever the objective, maximum transmission is detected in the blue range (448 nm, 488 nm) of the WLL (Figure S2). Whatever the laser line is, 86x objective displayed higher capacities of transmission compared to 93× and 100× (Figure S2). In particular, red light is better transmitted (+10–15%) through 86× compared to 93× or 100× (Figure S2).

**Figure 1:**
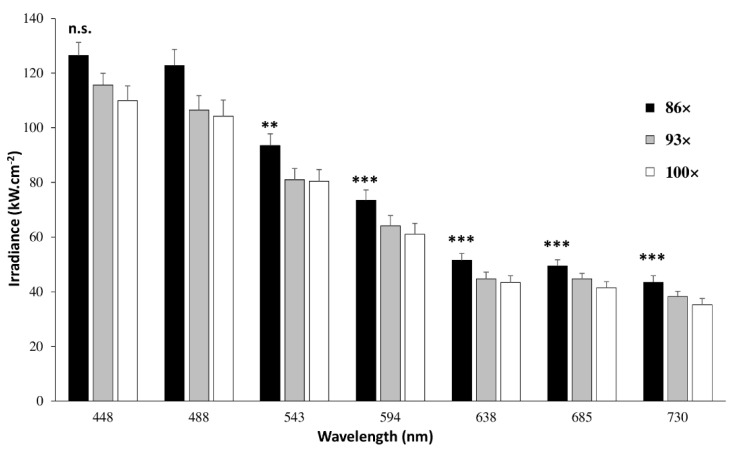
Irradiance of WLL lines through 86×, 93× and 100× objectives. Thanks to LAS-X software, laser power at 448, 488, 543, 594, 638, 685 and 730 nm was set at 47 µW and measured through an optical power meter integrated in Argo-POWER slide. By considering wavelength (nm) and numerical aperture (unitless), irradiance values (kW.cm^−2^) were calculated and directly read on Daybook 3 software from Argolight. Each value represents the mean (±SEM) of three independent experiments. Significant irradiance differences between WLL lines (n.s.; ** *p* < 0.01; *** *p* < 0.001 vs 488 nm) were determined for 86× using an ANOVA test followed by a Tukey–Kramer multiple comparison test.

Irradiance, or surface power density, is an indicator of how much light a living cell receives on a given surface. At the maximum power of each laser line, the maximum irradiance (228 kW.cm^−2^) with 86x was measured at 543 nm and the minimum at 730 nm (68 kW.cm^−2^) (Figure S3). Measurements at constant laser power (47 µW) revealed the lowest values of irradiance (35–55 kW.cm^−2^) within the red spectral range (638 nm, 685 nm and 730 nm, Figure 1), whatever the objective.

**Figure 2:**
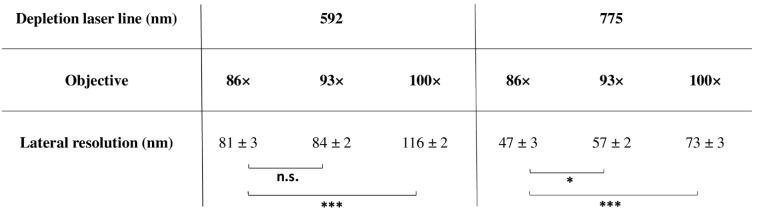
Lateral resolution through 86×, 93× and 100× objectives in stimulated emission depletion (STED) nanoscopy. Fluorescent Yellow-Green and Dark-Red beads (40 nm) were imaged through STED nanoscopy with a 592-nm and 775-nm depletion laser, respectively. Full Width Half Maximum (FWHM) measurement of the bead fluorescence signal with ImageJ led to determination of 86×, 93× and 100× lateral resolution. Each value represents the mean (±SEM) of minimum 10 independent experiments. n.s.; * *p* < 0.05; *** *p* < 0.001 vs 86× using an ANOVA test followed by a Tukey–Kramer multiple comparison test.

Increasing resolution through STED nanoscopy during live-cell imaging is generally challenging because of the high energy of the depletion laser. Whatever the objective, an increase in depletion laser power led to a linear increase in irradiance (Figure S4). Respective slopes characterizing 592-nm and 775-nm irradiance (28 vs 12) were dramatically different and maximum irradiance was detected for 100× objective (Figure S4). Fluorescent bead imaging through STED nanoscopy along the XY axis enabled the experimental determination of lateral resolution (Figure 2). At both 592 nm and 775 nm, the 86× objective displayed better lateral resolution as compared to 93× and 100× (Figure 2). In addition, the FLIM-STED approach with only 20% of 775-nm STED laser depletion power induced a significant improvement of lateral resolution (from 233 ± 10 nm to 54 ± 2 nm, *** *p* < 0.001) (Figure S5).

Characterization of the uniformity of the illumination field is necessary to avoid any misinterpretation in the intensity of fluorescence signals within the observation field. At zoom 0.75 and with a solution of Rhodamine 800, uniformity at 638 nm was only 49.3 with 86× objective (Figure S6A). An optical zoom of four dramatically increased uniformity to 91.3, leading to a homogeneous illumination field (Figure S6B). In addition, uniformity of the field of illumination obtained through 86× at zoom 0.75 was significantly better than was observed through 93× and 100× objectives (Figure S6C).

Taken together, the data suggest that homogeneous illumination of living cells with red/near-infrared wavelengths through an 86× objective with good transmission capacity will cause less phototoxic damage compared to the blue-green range of the light spectrum. These data also indicate that a 775-nm depletion laser should be used preferentially to reduce light exposure during STED and FLIM-STED nanoscopy of living cells.

**Figure 3:**
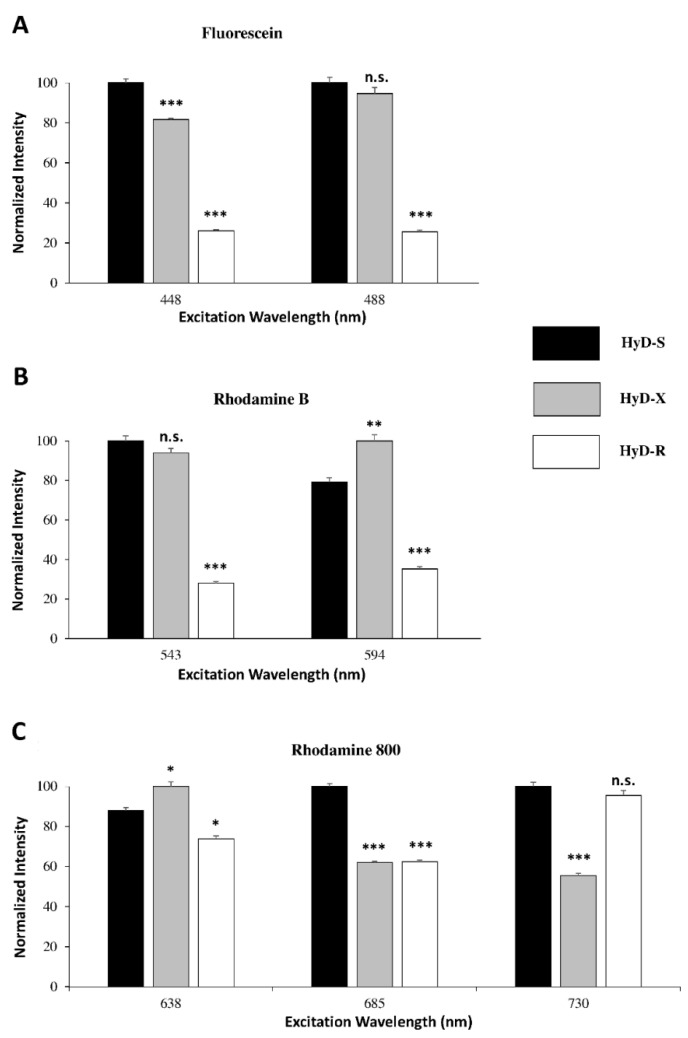
Spectral sensitivity of type S, X and R hybrid detectors (HyD-S, HyD-X and HyD-R). Spectral sensitivities of the HyDs were obtained by measuring the fluorescent intensity of fluorochrome solutions when excited at 448, 488, 543, 594, 638, 685 and 730 nm. (**A**) For the Blue-Green spectrum range, fluorescein solution was excited at 448 nm and 488 nm and fluorescence signal was collected at 460–510 nm and at 500–550 nm, respectively. (**B**) For the Orange-Red spectrum range, rhodamine B solution was excited at 543 nm and 594 nm and fluorescence signal was collected at 560–610 nm and 605–655 nm, respectively. (**C**) For near-infrared spectrum ranges, rhodamine 800 solution was excited at 638 nm, 685nm and 730 nm and fluorescence signal was collected at 650–700 nm, 695–745 nm and 740–790 nm, respectively. Each value represents the mean (±SEM) of three independent experiments. Significant spectral sensitivity between HyDs (n.s.; * *p* < 0.05; ** *p* < 0.01; *** *p* < 0.001 vs HyD-S) were determined using an ANOVA test followed by a Tukey–Kramer multiple comparison test.

The last generation of hybrid detectors known as type S (HyD-S), type X (HyD-X) and type R (HyD-R) have been characterized with regards to their spectral sensitivity. Fluorescein, rhodamine B and rhodamine 800 solutions were excited at 448/488 nm, 543/594 nm and 638/685/730 nm, respectively, and their fluorescence signal intensities measured by HyDs were compared. The HyD-S detector was broadly sensitive at the blue/green/orange spectrum range as well as within the near-infrared region (Figure 3). As compared to other detectors, HyD-X was more sensitive in the red spectrum range (Figure 3B). In contrast, HyD-R started to display interesting detection characteristics in the near-infrared spectrum (Figure 3C). Since τ separation represents an alternative strategy to spectral separation during multilabelling experiments, fine determination of fluorescence lifetime is necessary. The fluorescence lifetime reference values of fluorescein, rhodamine B and rhodamine 800 have been determined through activation of HyD-X and fast integrated FLIM module (Figure S7). Fluorochrome solutions were prepared in PBS. In these conditions, the fluorescence lifetimes of fluorescein, rhodamine B and rhodamine 800 were respectively 3.55 ± 0.04 ns (at pH = 7.5), 1.47 ± 0.06 ns (at pH = 6.5) and 0.7 ± 0.01 ns (at pH = 6.75) (Figure S7).

### 2.2. Optimization of Live-Cell Imaging through Confocal Microscopy and FLIM

#### 2.2.1. Intensity Images through Confocal Microscopy and Detector-Related/Phasor-Plot τ Separation

**Figure 4:**
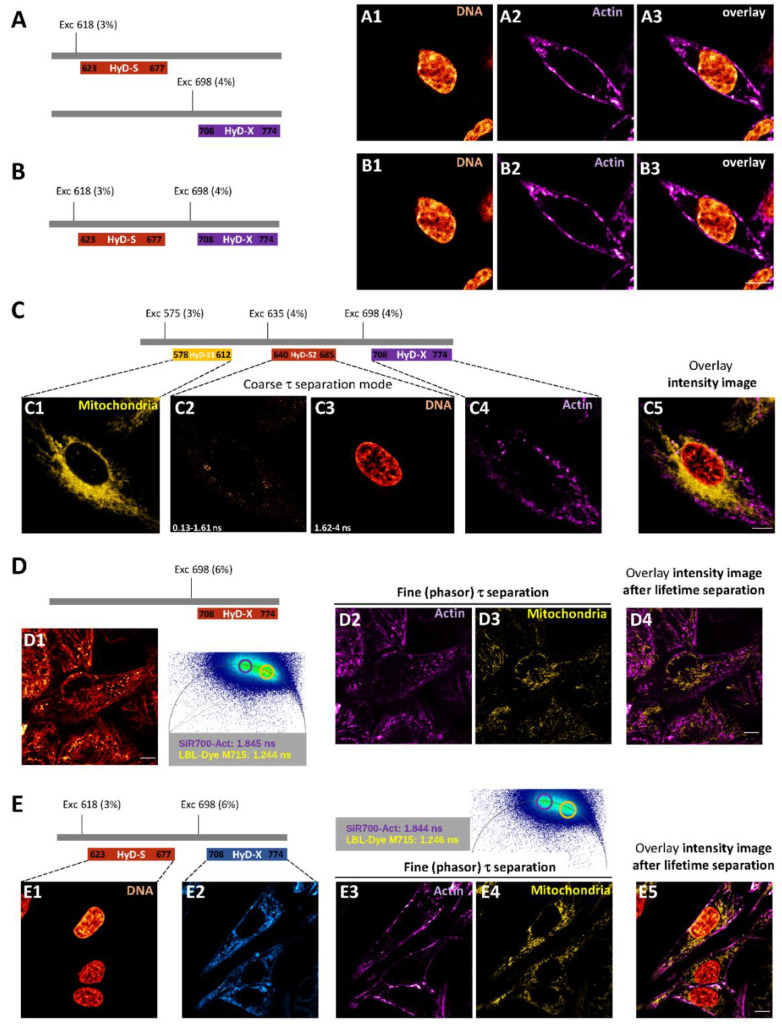
Input of coarse and fine fluorescence lifetime (τ) separation in organic red/near-infrared multiplexing with living H28 cells. Confocal imaging of H28 living cells stained with SPY620-DNA (red hot) and SiR700-Actin (magenta hot) in sequential (**A**) or simultaneous (**B**) mode. (**C**) Confocal imaging of triple-stained H28 cells in simultaneous mode. MitoTracker red (ex 594 nm—HyD-S1; (**C1**)), SPY620-DNA dye (ex 618 nm—HyD-S2; (**C2**,**C3**)) with coarse τ separation and SiR700-Actin dye (exc 698 nm—HyD-X; (**C4**)). (**D1**) Confocal imaging of H28 living cells stained with SiR700-Actin and LBL-Dye M715. (**D2,D3**) Fine τ separation of fluorescence lifetime components through phasor plot analysis after activation of FAst Lifetime CONtrast (FALCON) module and overlay of resulting imaging (**D4**). (**E**) Confocal imaging of triple-stained H28 cells in simultaneous mode. SPY620-DNA (ex 618 nm—HyD-S; (**E1**)) and SiR700-Actin with LBL-Dye M715 (ex 698 nm—HyD-X; (**E2**)) with fine τ separation through phasor plot analysis after activation of FALCON module (**E3**,**E4**) and overlay of resulting imaging (**E5**). Scale bars 10 µm.

Reducing light excitation and acquisition time is always a challenge when performing live-cell multiplexing through confocal microscopy. For spectrally distant fluorochromes, a double-labeling experiment is traditionally carried out through sequential detection in order to avoid any overlapping, as shown in Figure 4A for SPY620-DNA and SiR700-Actin H28 labeled cells. Due to HyD-S and HyD-X sensitivity, 86× transmission, and low laser power (618 nm, 3% of AOTF; 698 nm, 4% of AOTF) image acquisition through simultaneous detection could be performed, while acquisition time was reduced twofold (Figure 4B). A triple labelling with MitoTracker Red, SPY620-DNA and SiR700-Actin was even possible via simultaneous detection by activation of a second HyD-S (Figure 4C). In this case, slight shifts in SPY620-DNA excitation (from 618 to 635 nm) and detection (from 623–677 nm to 640–685 nm) together with activation of detector-related τ separation (i.e., coarse τ separation) were necessary to properly distinguish fluorochromes and to suppress background noise (Figure 4C). Due to the low light exposure and fast acquisition time, a combination of z-stack and time-lapse lasting up to 30 min could be performed (Video S1). For close fluorochromes such as SiR700-Actin and mitochondrial marker LBL-Dye M715, spectral separation could not be considered. Due to partial common absorption spectrum, both fluorochromes were excited and detected through single excitation wavelength (698 nm, 6% AOTF) and single HyD-X (708–774 nm) (Figure 4(D1)). Activation of the fast integrated FLIM module and phasor plot analysis enabled determination of LBL-Dye M715 (1.244 ns) and SiR700-Actin (1.845 ns) fluorescence lifetime (Figure 4(D1)). Based on experimental fluorescence lifetime values, further processing through fine τ separation (Figure 4(D2−D4)) led to separation of the LBL-Dye M715 and SiR700-Actin signals and to an intensity overlay image. For triple labelling with SPY620-DNA, SiR700-Actin and LBL-Dye M715, simultaneous activation of HyD-S and HyD-X combined with fine τ separation enabled rapid acquisition and an intensity overlay image after fluorescence lifetime separation (Figure 4E).

#### 2.2.2. Fluorescence Lifetime Images through Fitting Analysis, Phasor-Plot or Phasor-Plot τ Separation

**Figure 5:**
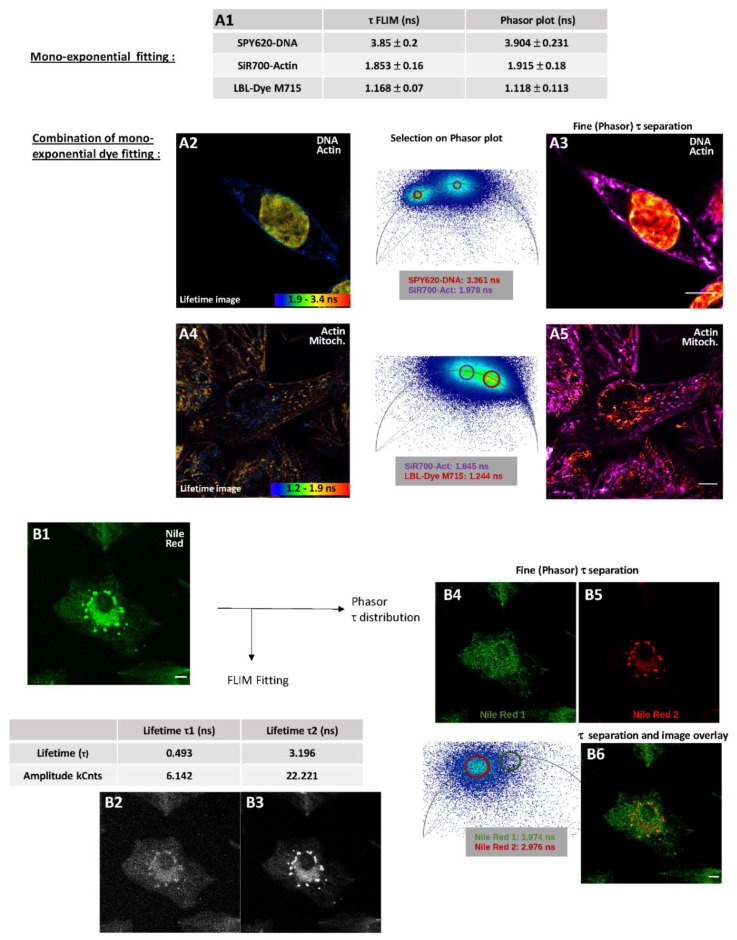
Input of fitting and phasor plot analyses in fluorescence lifetime microscopy (FLIM) of living H28 cells labeled with red/near-infrared dyes. (**A1**) Determination of fluorescent lifetimes for SPY620-DNA, SiR700-Actin and LBL-Dye M715 by using fitting and phasor plot analyses. FLIM (**A2**) and fine τ separation (**A3**) images of SPY620-DNA- and SiR700-Actin-labeled H28 cells (ex 618 nm, 5%; em 623–770 nm, HyD-X). FLIM (**A4**) and fine τ separation (**A5**) images of SiR700-Actin and LBL-Dye M715 labeled H28 cells (ex 698 nm, 6%; em 708–774 nm HyD-X). (**B1**) Confocal imaging (signal intensity) of Nile Red-labeled H28 cells (ex 552 nm, 3%; em 560–650 nm, HyD-X). Separated images through (**B2**,**B3**) fitting analysis or (**B4**,**B5**) fine τ separation (phasor plot) of Nile Red-labeled H28 cells after activation of FALCON module. Overlay of resulting fine τ separation images (**B6**). Scale bars 10 µm.

From the fast integrated FLIM module, fluorescence lifetime data could be processed differentially through the fitting and phasor plot approaches. Therefore, the lifetimes of SPY620-DNA, SiR700-Actin and LBL-Dye M715 were consequently obtained from mono-labeled-H28 cells and then compared. Application of mono-exponential fitting with good χ^2^ values revealed lifetimes of 3.85 ± 0.2 ns, 1.853 ± 0.16 ns and 1.168 ± 0.07 ns for SPY620-DNA, SiR700-Actin and LBL-Dye M715, respectively (Figure 5(A1)). Illustration of the TCSPC curve and single-exponential fit together with the distribution of arrival times is presented for SPY620-DNA in Figure S8. Determination of fluorochrome lifetimes through selection in the phasor plot did not show any major differences (Figure 5(A1)). For double-labeled H28 cells with SPY620-DNA and SiR700-Actin two modes of representation are relevant, including a contrasted rainbow FLIM fitted image (Figure 5(A2)) and a phasor plot-based τ separation image (Figure 5(A3)). In contrast, rainbow FLIM representation did not clearly show SiR700-Actin and LBL-Dye M715 staining (Figure 5(A4)), while a phasor plot-based τ separation image (Figure 5(A5)) was more appropriate to illustrate double-labeled H28 cells.

**Figure 6:**
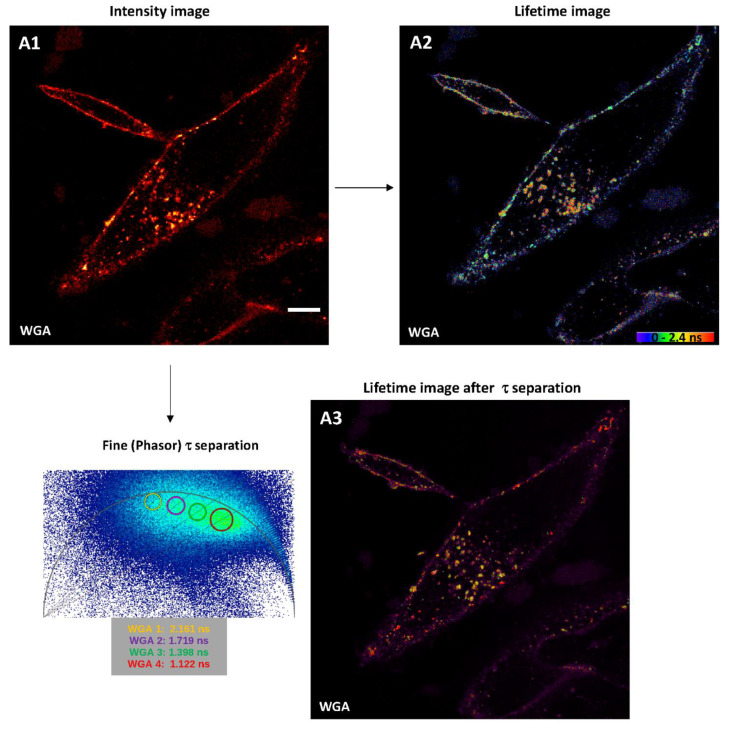
Input of fitting and phasor plot analyses in fluorescence lifetime microscopy (FLIM) of living H28 cells labeled with Alexa Fluor 633-WGA. (**A1**) Confocal imaging (signal intensity) of Alexa Fluor 633-WGA-labeled H28 cells (ex 633 nm, 3%; em 640–760 nm, HyD-X). FLIM (**A2**) and fine τ separation (**A3**) images of Alexa Fluor 633-WGA-labeled H28 cells. Scale bars 10 µm.

For dyes that may stain widely, lipids or membranes such as Nile Red or Alexa Fluor-coupled WGA, FLIM are also very helpful in order to discriminate complex microenvironmental variations during intracellular trafficking, endocytosis, or membrane recycling. When H28 cells are incubated with Nile Red for 10 min numerous intracellular structures are stained (Figure 5(B1)); however, these are poorly discriminated. When processed through multi-exponential fitting analysis, the very high χ^2^ value (~14) revealed two lifetimes (0.493 ns and 3.196 ns) that led to insufficient rendering when separated (Figure 5(B2,B3)). In addition, it cannot be excluded that the shorter lifetime component belongs to the instrument response function (IRF). When processed through phasor plot approach, a main lifetime component of Nile Red at 2.976 ns (Nile Red 2) was identified in living H28 cells as lipidic vesicles (Figure 5(B2)). Nile Red 1 selected a second minor lifetime component (1.974 ns) that could either correspond to background signal/IRF (Figure 5(B3)) and/or additional Nile-Red-positive elements within H28 cells (Figure 5(B4)). When H28 cells were incubated with Alexa Fluor 633-WGA, plasma membrane was rapidly stained while endocytosis led to a broad labeling of hardly-distinguishable vesicles over time (Figure 6(A1)). From genesis to trafficking, vesicle acidification is generally described as a marker of maturation; however, pH is also known to affect fluorescence lifetime. Compared to the intensity image, the rainbow phasor plot-lifetime image offered a lifetime distribution already within range of 0–2.4 ns (Figure 6(A2)). Application of τ separation on the phasor plot lifetime image through manual selection enhanced identification of categories of vesicles-like structures based on four different lifetimes, including 2.161 ns, 1.719 ns, 1.398 ns and 1.122 ns (Figure 6(A3)). By limiting light exposition time, the dynamic of τ-separated vesicles could be observed through mid-time-lapse experiments (Video S2).

### 2.3. One and Two-Color FLIM-STED Nanoscopy with Red/Near-Infrared-Labeled Living Cells

**Figure 7:**
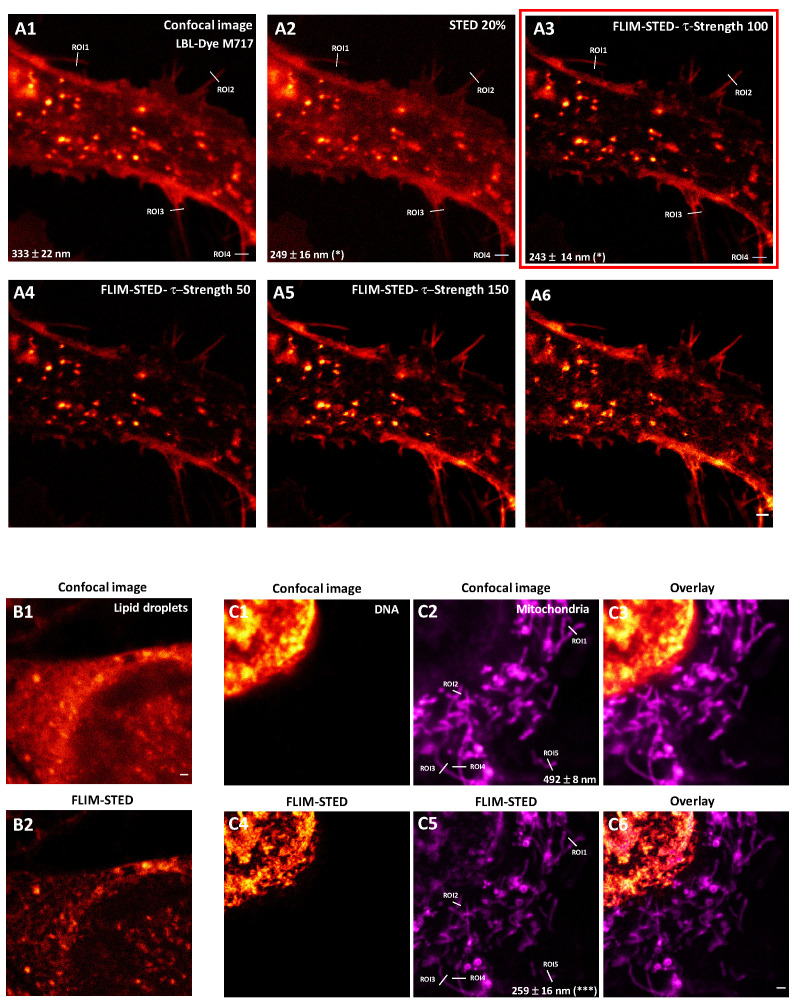
Input of FLIM in STED nanoscopy of living H28 cells labeled with red/near-infrared dyes. Confocal (**A1**) and STED (20% 775-nm depletion laser, (**A2**)) imaging of LBL-Dye M717-labeled H28 cells (ex 690 nm, 3%, HyD-X). (**A3**–**A6**) Impact of τ Strength factors (50–200) on STED imaging. Red box indicates best parameters configuration. Significant lateral resolution differences between imaging approaches (* *p* < 0.05 vs confocal approach) were determined using an ANOVA test followed by a Tukey–Kramer multiple comparison test. Confocal (**B1**) and FLIM-STED (100 τ Strength factor, (**B2**)) imaging of Nile Red-labeled H28 cells (ex 660 nm, 5%, HyD-X). Confocal (**C1**–**C3**) and FLIM-STED (100 τ Strength factor, (**C4**–**C6**)) imaging of SPY620-DNA-(ex 618 nm, 15%, em 625–670 nm, HyD-X) and LBL-Dye M715 (690 nm, 20%, em 700–750 nm, HyD-R)-labeled H28 cells in simultaneous mode. Significant lateral resolution differences between imaging approaches (*** *p* < 0.001 vs confocal approach) were determined using Student’s *t*-test. Scale bars 1 µm.

Combination of FLIM and STED approaches known as FLIM-STED imaging led to removal of uncorrelated STED process photons through background substraction and weighting of lifetimes (τ STED Strength). By taking advantage of lifetime data, live-cell STED imaging could consequently be achieved through reduction of light exposure (Figure S9). Additional signal smoothing through a wavelet filter (Denoising) could also be applied. Fixed Atto-647N-tubulin immuno-labeled H28 cells were used to test parameters for τ STED Strength and Denoising. Optimization of STED images (20% 775-nm depletion laser) was achieved with different τ STED Strength and Denoise parameters (Figure S9). For this experimental configuration, a τ STED Strength of 100 and Denoise of 50 induced a contrasted and resolved image (105 ± 10 nm compared to 266 ± 30 nm in confocal microscopy, *** *p* < 0.001). Since Atto-647N-labeled H28 cells were fixed, further resolution improvement could be obtained with 70% of the depletion laser (63 ± 3 nm) (Figure S9). Interestingly, a similar τ STED Strength factor and Denoise factor for FLIM-STED were applied for living H28 cells that were labeled with LBL-Dye M717 (Figure 7); in these conditions, contrast and lateral resolution were improved (from 333 ± 22 nm to 243 ± 14 nm, * *p* < 0.05, Figure S10) with only 20% 775-nm laser (25.2 mW, irradiance = 25 MW.cm^−2^, Figure S4) (Figure 7(A2,A3)). Excessive τ STED Strength factor (150–200) should be carefully considered at the risk of image degradation (Figure 7(A5,A6)). Similar parameters were successively applied to Nile-Red labeled H28 living cells (Figure 7B). Furthermore, application of only 5% 775-nm depletion laser combined with denoise 50 and strength of 100 on SPY620-DNA- and LBL-Dye M715-labeled living H28 cells significantly improved (*** *p* < 0.001) lateral resolution, from 492 ± 8 nm (confocal approach) to 258 ± 16 nm (FLIM-STED approach) (Figure 7C, Figure S10). Consequently, short two-color FLIM-STED time-lapse could be performed due to reduction of light exposure (Video S3).

## 3. Discussion

In this study, we developed complementary strategies in order to optimize live-cell imaging based on the Probe–Sample–Instrument concept [1]. We focused on red/near-infrared dyes for multi-labeling because they are out of spectral range of cell autofluorescence and their excitation induces less scattering and less cytotoxicity. Considering multiplexing with red/near-infrared fluorochromes for live-cell imaging, we first determined the best instrumental configuration to reduce light exposure and consequently to preserve samples. Then, we proposed an agile method including coarse τ separation for confocal microscopy, fine τ separation for FLIM, and enhanced FLIM-STED nanoscopy to improve fluorescence signals separation, temporal and spatial resolutions. The detailed approaches together with examples of their applications should provide a comprehensive guide for researchers who are considering such experiments.

**Figure 8:**
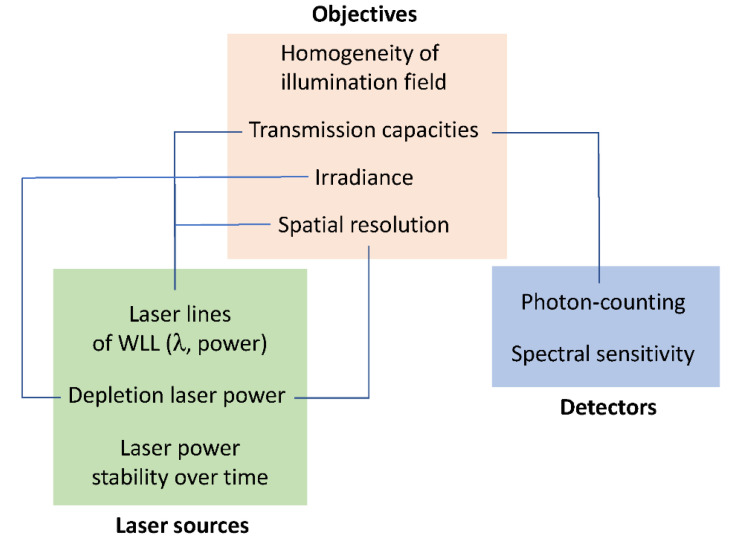
Instrumental characterization for optimization of live-cell imaging. Blue lines indicate relationships between key elements of advanced light microscopies.

Whatever the type of advanced light microscopy, there is a necessity to control instrumental performances to obtain robust, reproducible and comparable data [26,27,28,29,30]. By characterizing key elements, we aimed to limit the negative impact of light exposition through reduction of irradiance and exposure time (Figure 8). When considering the preservation of living cells, it should be noted that the nominal power of laser sources might be different [1] and filters, such as AOTF, might consequently be adjusted to limit light exposure. This is also the case for multi-line lasers, including the supercontinuum source type that we used in our experiments [31]. From selected WLL lines, the maximum power was measured in the orange range while blue and red wavelengths were less powerful. All lines were stable during short and mid periods, suggesting appropriate conditions for time-lapses. In the context of 2D cultured H28 cell imaging, high-resolution objectives including 86× (NA = 1.2, WD = 300 µm, water immersion), 93× (NA = 1.3, WD = 300 µm, glycerol immersion) and 100× (NA = 1.4, WD = 100 µm, oil immersion) were compared. The 86× objective offered the best uniformity of illumination field. From the original image center, a minimum zoom factor of 1.5 could be applied to reduce lateral deviation and adjust pixel size. The 86× objective also revealed higher capacities of transmission in the red/near-infrared range and higher lateral resolution through STED nanoscopy. Degradation of transmission and spatial resolution may occur overtime due to accidental impact, dirt or the effects of prolonged high laser power [26,27].

Taken together, this suggests that 86× would be able to better transmit low fluorescent signals as compared to other objectives. Since 86× is also a water immersion objective, the refractive index will be very similar in both immersion and culture medium-promoting fluorescence transmission. In addition, combination of 86× and red/near-infrared laser lines enabled the lowest irradiance values. During STED nanoscopy, pulsed 775-nm laser that depleted red/near-infrared dyes induced less irradiance than continuous (CW) 592-nm laser. In addition, the average power required with a pulsed STED laser is much lower than for a CW STED one to obtain the same lateral resolution [32], and since fluorophores are allowed to relax from triplet states before being re-excited, photobleaching is reduced [33]. Consequently, we used an 86x water immersion objective to image living cells labeled with red/near-infrared dyes through confocal microscopy, FLIM, and FLIM-STED nanoscopy.

**Figure 9:**
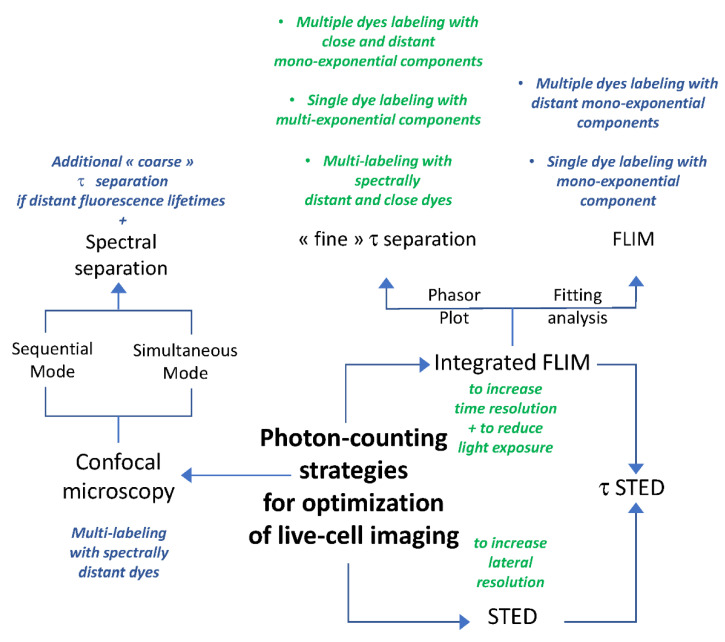
Photon-counting strategies for optimization of live-cell imaging. Agile method for selecting appropriate optimization of live-cell imaging with red/near-infrared dyes.

Optimization of live-cell imaging through fluorescence lifetime-based strategies is highly dependent on high-speed photon counting (Figure 9). In our study, experiments were performed through HyD-S, HyD-X and HyD-R that enabled detection of fluorescence signals up to 850 nm. HyD-S is based on silicon multi-pixel photon counter technology, in which incident photons are spread on an array of independent avalanche photodiodes [19,34]. HyD-S had a rather good sensitivity all over the spectrum in this study. HyD-X and HyD-R are based on hybrid detector technology with a gallium arsenide phosphide photocathode coupled to an avalanche diode [19,35]. HyD-X had better sensitivity in the red spectrum in this study. HyD-R is especially tailored for the near-infrared region of the spectrum due to an extended red gallium arsenide phosphide photocathode front plate [36]. All detectors have a very short dead time, below 1.5 ns, during which the detector is unable to detect a second photon. To gain time, coarse τ separation through detector-related τ separation (also known as TauContrast with HyDs) could be performed; however, post-acquisition processing is not possible since pixel lifetime values are not retained during acquisition [37]. Due to temporal and physical characteristics, HyD-X was selected for fluorescence lifetime measurements through fast integrated FLIM module and calibrated for phasor plot analysis. When a second detector was required, as presented in Figure 7C, HyD-R could also be used for double-labeling experiments. In contrast to slow TCSPC, field-programmable gate array (FGPA) in the FLIM module enables ”fast FLIM” by directly measuring differences in arrival times between detection and excitation pulses [18,36]. In these conditions, fine τ separation is achieved and can be applied with most fluorescence dyes during live-cell imaging [18]. Reference values of fluorescence lifetime for fluorescein, rhodamine B and rhodamine 800 measured with our FLIM module were consistent with data from the literature [16]. Therefore, fast electronics including readout circuitry and sensitive spectral detectors combined with algorithms for data acquisition and analysis offer new perspectives for multiplexing and lifetime acquisition in living cells. Nevertheless, line repetition is necessary in order to accumulate enough photon budget when performing live-cell FLIM.

As proposed in Figure 9, signal detection through photon counting mode reduced acquisition time and enabled complementary post-acquisition processes, leading to wide data exploitation. Here, we have tested different modes of acquisition in order to optimize red/near-infrared-labeled cell imaging. As mentioned previously, coarse τ separation can improve multi-labeled live-cell confocal imaging if dyes display distant spectral and lifetime characteristics. With spectrally close fluorochromes or with close lifetimes, HyD-X was the sole detector used due to its characteristics and its calibration with the FLIM module. Historically, FLIM data were processed by fitting the decay of each pixel using one or several exponentials, leading to identification of the decay times and amplitudes of components [16,17,19]. This approach is not simple due to the complexity of decay patterns and information extraction. Alternatively, the phasor method vectorially transforms decay times, and each pixel in the FLIM image corresponds to a point in the phasor plot [38]. Consequently, the phasor plot, which is represented as a half circle (also known as a universal circle), with longest and shortest lifetimes, respectively, on the left and right parts, provides a 2D graphical view of lifetime distributions [39]. Mono-exponential lifetimes lie on the universal circle line. Multi-exponential lifetimes are found inside the universal circle as a combination of their mono-exponential lifetime components. Therefore, the phasor approach simplifies the analysis of FLIM images, and application of appropriate regions of interest within the universal circle enables fine τ separation [39].

From this work, we proposed an agile method that could therefore be applied to determine appropriate strategies for advanced live-cell imaging (Figure 9). When living cells are labeled with a single dye, there are two possibilities depending on the number of fluorescence lifetime components. With one component, both fitting and phasor plot analysis could be applied, leading to similar fitted and lifetime images, respectively. With multiple components, only the phasor plot analysis and τ distribution in large scaling are relevant, as shown for Nile Red- and Alexa Fluor 633 WGA-labeled cells for separated lifetimes imaging. Additional adjustments targeting organelles within living cells may enhance lifetime separation as for separation of Alexa Fluor 633 WGA-labeled vesicle-like structures (Figure 9). When living cells are labeled with two dyes, each with one component, there are also two possibilities depending on the proximity of fluorescence lifetimes. If dyes display distant fluorescence lifetimes, both fitting and phasor plot analysis can be applied, leading to similar fitted and lifetime images, respectively (Figure 9). In contrast, if dyes display close fluorescence lifetimes (only different by several hundred ps), only the phasor plot analysis and τ distribution are relevant, as shown for SiR700-Actin and LBL-Dye M715. When H28 cells were labeled with three dyes, a combination of spectral separation with either coarse or fine τ-separation could be applied, as shown for MitoTracker Red/SPY620-DNA/SiR700-Actin and for SPY620-DNA/SiR700-Actin/LBL-Dye M715. As an additional application, phasor plot analysis has been also considered for FLIM-FRET experiments with fluorescent proteins [40]. Further studies are required to demonstrate whether FLIM-FRET experiments could be performed with organic red/near-infrared fluorochromes.

Using conventional STED nanoscopy to image dynamic processes in living cells has been challenging, mainly due to low signal-to-noise ratio and to high light exposure, which may cause cell damage [23]. Two-colour live-cell STED imaging with organic SiR and Atto dyes has been previously performed with a custom-built STED nanoscope [25]. In this work, we studied the advantages of STED and FLIM combination through a commercial STED nanoscope. We first showed that the harmful effects of light exposure are reduced by the recently available 775-nm depletion laser line, which is much less powerful, and by low irradiance through an 86x objective with red/near-infrared lines. Enhancement of STED performance through fluorescence lifetime component exploitation has been previously demonstrated [22,41]. During the STED process, a depletion laser induces a de-excitation of fluorophores to return to S0 via stimulated emission, and consequently modifies fluorescence lifetimes [24]. Since the depletion STED beam has a donut shape, the shortest fluorescence lifetimes come from the periphery while the longest fluorescence lifetimes originate from the center. Therefore, due to the energy gradient of the STED beam geometry, a gradient of fluorescence lifetime can be observed. When a temporal window is applied in a gated-STED mode, photons with early arrival times are excluded. This approach increased the resolution; however, there was a loss of information that led to a low SNR [22,41]. More recently, maximum (donut periphery) and minimum (donut center) STED energies have been mapped in a phasor plot and used to define a STED trajectory containing all the photons of the STED process [42]. In this context, application of a complex wavelet filter resulted in removal of fluorescence lifetimes uncorrelated to the STED process, and consequently in improvement of resolution through exploitation of the fluorescence lifetime gradient along the STED trajectory [43]. Here, we showed a STED effect of 20% 775-nm depletion laser (irradiance 25.3 MW.cm^−2^) on H28 cells labeled with LBL-Dye M717, SPY620-DNA or Nile Red. By adjusting background substraction, lifetime weighting, and signal smoothing, single and double-labeled living H28 cells were imaged through FLIM-STED nanoscopy including two-colour time-lapse. Therefore, combining optical signals from STED with the physical information from the fluorescence lifetime, also described as FLIM-STED nanoscopy, offers new perspectives to increase resolution and eliminate background noise together with limited photon numbers and reduced light exposure.

## 4. Materials and Methods

### 4.1. Instrumental Characterization

#### 4.1.1. Confocal, FLIM, STED Microscope

- Confocal microscopy: A commercial inverted confocal laser scanning microscope (STELLARIS 8, Leica Microsystems, Nanterre, France) was equipped with a white light laser (440–790 nm). From Leica Microsystems (Nanterre, France) an 86× objective (NA = 1.20, water immersion, WD = 300 µm), 93× objective (NA = 1.30, glycerol immersion, WD = 300 µm) and 100× objective (NA = 1.40, oil immersion, WD = 100 µm) were used in this study. Fluorescence signals were detected in photon counting mode through four new generation Power HyD detectors including two Power HyD-S (Silicon Multi-Pixel Photon Counter; HyD-S1 and HyD-S2), one Power HyD-X (GaAsP Hybrid) and one Power HyD-R (Extended red GaAsP Hybrid). For image acquisition, appropriate zoom factor and pixel size were set in coherence with samples. A full bold line Okolab chamber (Ottaviano, Italy) installed on the inverted microscope stand was used to keep the temperature at 37 °C during image acquisition.
- FLIM: Fluorescence Lifetime imaging was performed with a fully fast integrated FLIM module, the so called FAst Lifetime CONtrast (FALCON, Leica Microsystems, Nanterre, France). FLIM images were acquired with accumulation to obtain an appropriate photon budget for phasor plot or fitting analysis.
- STED nanoscopy: A 2D STED module (Leica Microsystems, Nanterre France) installed on a STELLARIS 8 confocal base integrating two depletion lasers was used in this study. A 592-nm continuous depletion laser was dedicated to fluorochromes, with emission ranging from 470 nm to 550 nm. A 775-nm pulsed depletion laser was dedicated to fluorochromes with emission between 580 nm and 750 nm. STED images were acquired with appropriate zoom factor to ideally achieve a pixel size of 20 nm as well as frame accumulation and optimized gating for labeled samples.

#### 4.1.2. Laser Power, Irradiance and Transmission

Laser power, irradiance and transmission data were obtained via an Argo-POWER slide (Argolight, Talence, France) which integrates an optical power meter. As for biological samples, Argo-POWER slide was positioned on a SuperZ-galvo motorized stage. Daybook 3 software (Argolight) controlled sensor parameters including laser wavelength (448 nm, 488 nm, 543 nm, 594 nm, 638 nm, 685 nm, 730 nm for WLL and 592 nm, 775 nm for depletion lasers), sampling period (1 s or 5 s), acquisition duration (30 s, 10 min or 1 h), type of illumination (point-scanning) and objective numerical aperture (NA). WLL and depletion lasers were warmed up for 1 h before any measurement. All values including average, standard deviation and deviation, stability, maximum and minimum were directly read on Daybook 3 software.

#### 4.1.3. Uniformity of Field Illumination

To determine uniformity of field illumination, three homogeneous fluorescent solutions were prepared with classical fluorochromes including fluorescein (ex 440–520 nm; em 450–550 nm), rhodamine B (ex 520–620 nm; em 530–650 nm) and rhodamine 800 (ex 630–750 nm; em 640–800 nm). Fluorescent stock solutions in the amount of 20 µL of 10^−3^ M were mixed with 300 µL of PBS/glycerol solution (50/50), dropped on 35-mm glass bottom dish (MatTek Corporation, Ashland, MA, USA) and sealed with a coverslip. Determination of field illumination uniformity at different laser lines was performed with fluorescent solutions, i.e., fluorescein (448 nm and 488 nm), rhodamine B (543 nm and 594 nm) and rhodamine 800 (638 nm, 685 nm and 730 nm), as well as through different objectives (86×, 93× and 100×). Fluorescence was detected through HyDs used in photon counting mode and laser power was adjusted to avoid signal saturation. Acquisitions were performed with a 512 × 512 image format, a 0.75 zoom factor, and a 400 Hz scan speed with 4-line and 8-frame averages. Images were analyzed through the ImageJ plugin MetroloJ-QC [44]; https://github.com/MontpellierRessourcesImagerie/MetroloJ_QC) (accessed on 7 October 2021).

#### 4.1.4. HyDs Sensitivity

HyD-S, HyD-X and HyD-R sensitivities were determined with three homogeneous fluorescent solutions of fluorescein, rhodamine B and rhodamine 800. HyDs were used in photon counting mode. To compare detectors, the laser power of WLL lines and the emission band were standardized for fluorescein (ex 448 and 488 nm; em 460–510 nm and 500–550 nm, respectively), rhodamine B (ex 543 and 594 nm; em 560–610 nm and 605–655 nm, respectively) and rhodamine 800 (ex 638, 685 and 730 nm; em 650–700 nm, 695–745 nm and 740–790 nm, respectively). Acquisitions were performed with a 512 × 512 image format, a 0.75 zoom factor, and a 400 Hz scan speed with 4 line and 8 frame averages. Mean signal intensity of the whole images (ImageJ) revealed HyD sensitivities.

#### 4.1.5. FLIM Calibration

Fluorescent solutions of fluorescein, rhodamine B and rhodamine 800 were also used for determination of reference values of fluorescence lifetime. FLIM images were acquired with a photon budget of 100 photons/pixel through the FALCON module and activation of HyD-X in photon counting mode. The laser power of WLL lines and the emission band were standardized for fluorescence lifetime measurement. The pH of fluorescent solutions was measured through an in-lab nano pH-meter (Mettler Toledo, Viroflay, France). Acquisitions were performed with a 512 × 512 image format, a 0.75 zoom factor and a 400 Hz scan. Fluorescence lifetime values were obtained through fitting analysis.

#### 4.1.6. Super-Resolution and Objectives

Bead (FluospheresTM Carboxylate-Modified Microspheres, F8795 and F8789, Thermo Fisher Scientific, Illkirch-Graffenstaden, France) imaging was used to compare objective performances through STED nanoscopy. Bead diluted solution (~1.42 × 10^9^ beads/mL) was obtained through three successive dilutions of stock solution (1/100 each); 10 µL of diluted bead solution was dropped on a glass slide, successively covered by 20µL of PBS/glycerol (50/50) and a grade 1.5 coverslip. Yellow-Green beads (40 nm) were excited at 500 nm (WLL, 25 % laser power) and depleted with 60% of a 592-nm STED laser. An HyD-S in photon counting mode was used to detect fluorescence emissions from 505 to 555 nm. For STED imaging, Dark-Red beads (40 nm) were excited at 660 nm (WLL, 100% laser power) and depleted with 60% of a 775-nm STED laser. The HyD-X in photon counting mode was used to detect fluorescence emissions from 665 to 735 nm. Acquisitions were performed with a 1024 × 1024 image format, an objective-dependent zoom (86×, zoom = 13.22; 93×, zoom = 12.22; and 100×, zoom = 11.36) for a 10-nm pixel size, and line and frame accumulations. For FLIM-STED imaging, Dark-Red beads (40 nm) were excited at 660 nm (WLL, 100% laser power) and depleted with 20% of a 775-nm STED laser. HyD-X in photon counting mode was used to detect fluorescence emission from 665 to 735 nm. Acquisitions were performed with a 1024 × 1024 image format, a scan speed at 100 Hz, an 86× objective (zoom = 13.22) for a 10-nm pixel size, and line repetition (2) through FALCON module. Full Width Half Maximum (FWHM) measurement of bead fluorescence signal with ImageJ [45] led to determination of 86×, 93× and 100× lateral resolution.

### 4.2. Cell Culture

Purchased from American Tissue Culture Collection (ATCC), NCI-H28 (RRID:CVCL_1555) cells (a cell line from human malignant pleural mesothelioma) were cultured in Roswell Park Memorial Institute-1640 (RPMI-1640) (Thermo Fisher Scientific, Illkirch-Graffenstaden, France) medium supplemented with 2 mM of L-glutamine, 10% heat-inactivated fetal bovine serum, 100 U/mL penicillin, 100 μg/mL streptomycin (Thermo Fisher Scientific, Illkirch-Graffenstaden, France), as previously described [46]. The H28 cell line had been authenticated using short tandem repeat profiling within the last three years. Cultured cells were incubated at 37 °C in a humidified atmosphere with 5% CO_2_. For imaging experiments, H28 cells were plated at a density of 0.4 × 10^4^ cells per cm^2^ on 35-mm glass bottom microwell dishes (MatTek Corporation, Ashland, MA, USA). All experiments were performed with mycoplasma-free cells.

### 4.3. Cell Labeling

For live-cell imaging, H28 cells were labelled at 37 °C/5% CO_2_ with single or multiple dyes including: SPY620-DNA (0.5 µM) and/or SiR700-Actin (0.5 µM) together with verapamil (10 µM) for 1 hr (Tebu, Le Perray-en-Yvelines, France); Red MitoTracker (0.2 µM) for 30 min (Thermo Fisher Scientific); LBL-Dye M715 (2 µg/mL) or LBL-Dye M717 (1.8 µg/mL) for 30 min (Proimaging, Paris, France); Nile Red ultra-pure (1 µM) for 10 min (Enzo, Villeurbanne, France); or Alexa Fluor 633-conjugated WGA (1 μg/mL) for 15 min (Thermo Fisher Scientific, Illkirch-Graffenstaden, France).

For tubulin-staining, H28 cells were fixed with 4% paraformaldehyde for 5 min. After 30 min exposure to 1% bovine serum albumin (BSA) in phosphate buffered saline (PBS), cells were incubated at 4 °C overnight with a monoclonal antibody directed against tubulin (1:1000, Merck, Fontenay-sous-Bois, France) in PBS supplemented with 1% BSA and 0.3% Triton X-100. Cells were washed by PBS three times for 5 min each and then incubated for 2 h at room temperature with Atto-647N-conjugated goat anti-mouse antibodies (Merck, Fontenay-sous-Bois, France) diluted 1:400 in PBS supplemented with 1% BSA and 0.3% Triton X-100. Finally, cells were washed by PBS three times for 5 min each and kept in PBS for observation.

### 4.4. Confocal Imaging of Red/Near-Infrared Labeled Live-H28 Cells and Coarse τ Separation

Confocal imaging of multi-labeled living H28 cells (SPY620-DNA, SiR700-Actin, Red MitoTracker) was performed with 575 nm, 618 nm, 635 nm, and 698 nm WLL lines (3–4% AOTF) for dye excitation and HyD-S and HyD-X for fluorescence collection through 86× (NA = 1.2) objective. A conventional scanner (400 Hz, 1024 × 1024, line average = 1, 2.58 s per frame) and Airy 1 pinhole were used. For triple-labeled cells, time-lapse was obtained at a frequency of one stack (z1 to z3, total thickness of 0.71 µm) every 20 s during 30 min. Images were processed through ImageJ for maximum projection and overlay.

### 4.5. FLIM Imaging of Red/Near-Infrared Labeled Live-H28 Cells and Phasor Plot Analysis

Confocal imaging of mono- or multi-labeled living H28 cells (SPY620-DNA, SiR700-Actin, LBL-Dye M715, Nile Red, Alexa Fluor 633-conjugated WGA) was performed with 552 nm, 618 nm, 633 nm, and 698 nm WLL lines (3–6% AOTF) for dye excitation and with HyD-X for fluorescence collection through 86× (NA = 1.2) objective. A conventional scanner (400 Hz, 1024 × 1024) and Airy 1 pinhole were used. FLIM module, line repetition 3, was activated for fluorescence lifetime determination. For Alexa Fluor 633-conjugated WGA-labeled cells, time-lapse was obtained at a frequency of one frame (line repetition 1) every 5 s during 5 min.

### 4.6. STED and FLIM-STED Imaging of Fixed and Live-H28 Cells

STED imaging of mono-labeled H28 cells was performed with 647 nm, 660 nm or 690 nm WLL lines for Atto-647N, Nile Red or LBL-Dye M717 excitation respectively, 775-nm laser for depletion, and HyD-X for fluorescence collection through an 86× (NA = 1.2) objective. STED imaging of double-labeled living H28 cells was performed simultaneously with 618 nm and 690 nm WLL lines for SPY620-DNA and LBL-Dye M715 excitation, 775-nm 5% laser for depletion and HyD-X and HyD-R for respective fluorescence collection through an 86× (NA = 1.2) objective. A conventional scanner (400 Hz, 1024 × 1024) and Airy 1 pinhole were used. A zoom of 6.65 was applied to obtain a pixel size of 19.87 nm. Gating between 0.3–1 ns to 6 ns was activated. A three- to six-line repetition was necessary to collect appropriate photon budget for FLIM-STED imaging. Application of 100 τ Strength filter and 50 denoise parameters were applied. For FLIM-STED imaging of double-labeled cells over time in a simultaneous mode, resonant scanner (8000 Hz), HyD-X and HyD-R were activated while image format was reduced to 512 × 512. Images were obtained at a frequency of one frame (line repetition 12 for accumulation) every 0.78 s during 50 s.

### 4.7. Statistical Analysis

All values are expressed as means ± SEM. Statistical analysis was performed using the GraphPad Prism 4 software (GraphPad Software Inc., San Diego, CA, USA) and a one-way analysis of variance (ANOVA) with a Tukey–Kramer multiple comparisons test or Student’s *t*-test.

## 5. Conclusions

In this study, we focused on 2D cultured cells labeled with organic red/near-infrared dyes, and described the advantages of appropriate instrumental configuration for advanced live-cell imaging according to the Probe–Sample–Instrument concept. In this context, we proposed new methods to reduce the impact of light exposition through limitation of irradiance and exposure time. Depending on spectral and fluorescence lifetime characteristics and number of dyes, complementary strategies including τ separation for confocal microscopy, FLIM with fitting or phasor plot analysis, and τ separation for FLIM-STED nanoscopy were depicted for fast, robust, spatially and temporally resolved acquisitions. Taken together, we demonstrated that live-cell FLIM and live-cell STED nanoscopy were most accessible for researchers. Further applications will also be dependent on development of new bright dyes with distant fluorescence lifetimes and STED compatibility.

## Data Availability

Not applicable.

## Supplementary Materials

The following are available online at https://www.mdpi.com/article/10.3390/ijms222011092/s1.
