# Supplementary material for: Optimization of Advanced Live-Cell Imaging through Red/Near-Infrared Dye Labeling and Fluorescence Lifetime-Based Strategies
Source: Int J Mol Sci. 2021 Oct 14;22(20):11092. doi: 10.3390/ijms222011092 (PMC8537913; doi:10.3390/ijms222011092)
Supplement: Supplementary file 1 [file ijms-22-11092-s001.zip › supplementary/Suppl Table 1 Patient Summary.docx]

**Table S1 - Summary of patient cohort clinical parameters and longitudinal CTC enumeration.** On the CTC column, italicized counts refer to same sample as used for RNA-Seq. The number given refers to CD45-/ EpCAM+/ CK+/ DAPI+ CTCs.

^1^ = CD45-/ EpCAM-/ CK+/ DAPI+ CTCs detected. ^2^ = CD45-/ EpCAM +/ CK-/ DAPI+ CTCs detected.

| **Patient** | **ER Status** | **PR Status** | **ERBB2**  **Status** | **Diagnosis** | **Stage at Presentation** | **Years Since Primary Diagnosis** | **Years Since Diagnosis with Metastatic Disease** | **Metastatic Sites** | **Endocrine Therapy in Metastatic Setting** | **Chemotherapy in Metastatic Setting** | | **CTC # by Rarecyte**  **(CTC/ml blood)** |
| --- | --- | --- | --- | --- | --- | --- | --- | --- | --- | --- | --- | --- |
| **1** | neg | neg | neg | Invasive Ductal Adenocarcinoma | Stage IV  (De-novo metastatic) | N/A | 2 years | Brain  Bone  Liver  Lymph Nodes  Ascites | Letrozole  Anastrozole  Palbociclib | Paclitaxel  Capecitabine  Eribulin | | ***Baseline:***  ***136 CTCs***  3 months:  >15,000^1,2^ CTCs |
| **2** | pos  (primary)  pos  (liver) | pos    neg | neg    neg | Invasive Ductal Adenocarcinoma | Stage IV  (De-novo metastatic) | N/A | 10 years | Liver  Bone  Brain | Tamoxifen  Letrozole  Fulvestrant  Palbociclib | Docetaxel  Venetoclax  Cyclophosphamide | | ***Baseline:***  ***1 CTCs***  ***6 months:***  ***Not Done (SC)***  9 months:  12 CTCs |
| **3** | pos | pos | neg | Invasive Mammary Carcinoma | Stage II | 1 year | 1 year | Bone | Anastrozole  Ribociclib | N/A | | Baseline:  102 CTCs  ***3 months:***  ***3 CTCs*** |
| **4** | pos | pos | pos | Invasive Ductal Adenocarcinoma | Stage IIA | 4 years | 3 years | Liver | Letrozole  Palbociclib | Trastuzumab  Capecitabine  Tucatinib | | ***Baseline:***  ***0 CTC^1^***  3 months:  7 CTCs^1,2^  6 months  50 CTC^,1,2^ |
| **5** | Pos | Pos | Pos | Right breast invasive mucinous carcinoma | Surgery in the last year  stage II-A | 1 year | N/A | N/A | N/A | N/A | | ***Baseline:***  ***0 CTC***  6 months:  2 CTC ^2^ |
| **6** | Pos | Pos | Pos | Invasive ductal carcinoma |  |  | 10 years | Bone | Letrozole  Exemestane  fulvestrant | Herceptin  Kadcyla  pertuzumab | | ***Baseline:***  ***7 CTCs^1,2^*** |
| **7** | Pos | Pos | Pos | Invasive ductal adenocarcinoma | stage II-A  T1c N1 M0 | 8 years | 5 years | Bones | fulvestrant | Paclitaxel  Pertuzumab  trastuzumab | | ***Baseline:***  ***8 CTCs^1,2^***  ***3 month:***  ***78 CTC ^1,2^***  ***6 month:***  ***3 CTCs ^1,2^*** |
| **8** | pos | pos | neg | Invasive Ductal Adenocarcinoma | Stage IV  (De-novo metastatic) | N/A | 2 years | Lung | Letrozole  Anastrozole  Fulvestrant  Ribociclib  Elacestrant  Everolimus | N/A | ***Baseline:***  ***1 CTCs***  ***3 months:***  ***2 CTCs^2^***  6 months  0 CTC^,^ | |
| **9** | pos | Pos | Neg | Invasive ductal adenocarcinoma | Stage IV  (De-novo metastatic) |  | 5 years | Bone | Anastrozole  Ribociclib  Fulvestrant  tamoxifen | Paclitaxel  Eribulin  Carboplatin  gemcitabine | Baseline:  ***0 CTCs^1,2^*** | |
| **10** | Pos | Pos | Neg | Invasive ductal carcinoma | Surgery within 1 year  stage III-B | 1 year | 1 year | Lymph Nodes |  | Adriamycin  Cyclophosphamide  paclitaxel | ***Surgery:***  ***23 CTCs ^1,2^***  3 months:  0 CTCs^1,2^ | |
| **11** | Pos (primary)  Pos (Lymph node_ | Pos  Neg | Neg  Neg | Invasive lobular adenocarcinoma | Stage IV  (De-novo metastatic) |  | 3 years | Bone | Letrozole  palbociclib |  | ***Baseline:***  ***1 CTCs^1,2^***  3 months:  1 CTC^1,2^ | |
| **12** | Pos | Neg | pos | Invasive ductal adenocarcinoma | Stage IV  (De-novo metastatic) |  | 1 year | Lymph Node  Bone | Anastrozole  palbociclib | Pertuzumab  trastuzumab | ***Baseline:***  ***1 CTCs^2^***  3 months:  7 CTCs ^1,2^  6 months:  1 CTCs ^2^ | |
| **13** | Pos | Neg | Pos | Invasive ductal adenocarcinoma | No Surgery |  | 2 years | Liver  Lymph node | Palbociclib  letrozole | Herceptin  Docetaxel  Pertuzumab  trastuzumab deruxtecan | Baseline:  0 CTCs^1,2^  ***3 months:***  ***6 CTC ^1,2^***  6 months  2 CTCs ^1,2^ | |
| **14** | Pos | Neg | Neg | Invasive Ductal Adenocarcinoma | Stage I-A | 12 years | 2 years | Lung  Lymph Nodes  Bone | Fulvestrant | Docetaxel  Xeloda | ***Baseline:***  ***1 CTCs^1,2^***  3 months:  0 CTCs^2^  6 months  3 CTC^,1^ | |
| **15** | Pos | Neg | Neg |  | stage II | 9 years | 2 years | Liver, Bone, Lymph | Palbociclib |  | ***Baseline:***  ***3 CTCs^1,2^***  3 months:  0 CTCs^1,2^ | |
| **16** | Pos | Neg | Neg | Invasive ductal carcinoma | stage II-B | 4 years | 1 year | Liver, Lungs  Pancreas, lymph node, adrenal glands | Fulvestrant  Ribociclib  anastrozole | Docetaxel  Herceptin  Pertuzumab  paclitaxel | ***Baseline:***  ***4 CTCs^1,2^***  3 months:  0 CTCs^1,2^ | |
| **17** | Pos | Neg | Neg | Invasive ductal adenocarcinoma | stage I-A | 6 years | 4 years | Bone  liver | Fulvestrant  letrozole  palbociclib | Cisplatin  Gemcitabine  paclitaxel | Baseline:  5 CTCs^1,2^  ***3 months:***  ***ND***  6 months:  2 CTCs ^2^ | |
| **18** | Neg | Neg | Pos | Invasive ductal adenocarcinoma | Stage IV  (De-novo metastatic) |  | 4 years | Bone  Lung  Liver | palbociclib | Paclitaxel  Pertuzumab  trastuzumab | Baseline:  0 CTCs^1,2^  ***3 months:***  ***1 CTC***  6 months  29 CTCs^1,2^ | |
| **19** | Neg | Neg | Pos | Invasive ductal adenocarcinoma | Stage IV  (De-novo metastatic) |  | 2 years | Liver |  | Docetaxel  pertuzumab trastuzumab  paclitaxel | Baseline:  0 CTCs^1,^  ***3 months:***  ***0 CTC^1^*** | |
| **20** | Neg | Neg | Pos | Invasive ductal carcinoma | Stage II | 6 years | 2 years | Liver  Lymph node | palbociclib | Paclitaxel  Trastuzumab  Pertuzumab | ***Baseline:***  ***1 CTCs^1,2^***  3 months:  0 CTC ^,2^  6 months  0 CTC^1,2^ | |
| **21** | neg | neg | neg | Infiltrating ductal carcinoma | Surgery within 1 year | 2 years | N/A | N/A | N/A | N/A | Baseline:  ND  ***3 months:***  ***ND***  6 months:  4 CTC | |
